# Echocardiographic assessment of left atrial appendage morphology and function—an expert proposal by the German Working Group of Cardiovascular Ultrasound

**DOI:** 10.1007/s00392-024-02492-5

**Published:** 2024-08-28

**Authors:** Andreas Hagendorff, Stephan Stöbe, Andreas Helfen, Fabian Knebel, Ertunc Altiok, Stephan Beckmann, Tarek Bekfani, Thomas Binder, Aydan Ewers, Ali Hamadanchi, Henrik ten Freyhaus, Thomas Groscheck, Dariush Haghi, Jan Knierim, Sebastian Kruck, Karsten Lenk, Nicolas Merke, Dietrich Pfeiffer, Elena Romero Dorta, Tobias Ruf, Christoph Sinning, Nina C. Wunderlich, Roland Brandt, Sebastian Ewen

**Affiliations:** 1 Department of Cardiology, University Hospital Leipzig AöR, Leipzig, Germany; 2 Department of Kardiologie, Katholische St. Paulus Gesellschaft, St.-Marien-Hospital Lünen, Lünen, Germany; 3 Department of Internal Medicine II, Cardiology, Sana Klinikum Lichtenberg, Berlin, Germany; 4 Department of Cardiology, Angiology, and Intensive Medicine, University Hospital Aachen, Aachen, Germany; 5 Privatpraxis Kardiologie, Beckmann Ehlers Und Partner, Berlin-Grunewald, Germany; 6 Department of Cardiology and Angiology, University Hospital Magdeburg AöR, Magdeburg, Germany; 7 Department of Cardiology, University Hospital AKH, Vienna, Austria; 8 Department of Cardiology and Angiology, BG University Hospital Bergmannsheil, Bochum, Germany; 9 Department of Cardiology, University of Jena, Jena, Germany; 10 Department of Internal Medicine III, Cardiology, University of Cologne, Cologne, Germany; 11 Kardiologische Praxisklinik Ludwigshafen-Akademische Lehrpraxis of the University of Mannheim, Ludwigshafen, Germany; 12 Department of Internal Medicine and Cardiology, Paulinenkrankenhaus Berlin, Berlin, Germany; 13 Praxis Für Kardiologie Cardio Centrum Ludwigsburg, Ludwigsburg, Germany; 14 Department of Cardiothoracic and Vascular Surgery, Deutsches Herzzentrum Charité Berlin, Berlin, Germany; 15 Kardiologische Praxis Berlin (Adlershof), Berlin, Germany; 16 Department of Cardiology, Angiology and Intensive Care Medicine, University of Berlin, Deutsches Herzzentrum Charité Berlin, Campus Mitte, Berlin, Germany; 17 Department of Cardiology, Center of Cardiology, Heart Valve Center, University Medical Center Mainz, University of Mainz, Mainz, Germany; 18 Department of Cardiology, University Heart and Vascular Center Hamburg, German Centre of Cardiovascular Research (DZHK), Hamburg, Germany; 19 Department of Cardiology, Asklepios Klinik Langen, Langen, Germany; 20 Department of Cardiology, Kerckhoff Klinik GmbH, Bad Nauheim, Germany; 21 Department Cardiology and Intensive Care Medicine, Schwarzwald-Baar Klinik, Villingen-Schwenningen, Germany; 22 University Heart Center Freiburg, Bad Krozingen, Freiburg, Germany

**Keywords:** Atrial fibrillation, Echocardiography, Left atrial appendage, LAA morphology, LAA function, LAA intervention

## Abstract

The left atrial appendage is a blind ending cardiac structure prone to blood stasis due to its morphology. This structure is a preferred region of thrombogenesis in relation to reduced myocardial contractility of the atrial wall. Blood stasis occurs primarily in low flow conditions. One of the tasks of echocardiography is the analysis of morphology and function of the left atrial appendage. The detection of thrombi by echocardiography is difficult and must be carried out thoroughly and carefully to avoid potential complications—especially in the context of rhythm control. The assessment of thromboembolic risk, especially in patients with unknown and presumed atrial fibrillation is a second challenge by characterizing atrial function and flow conditions in the left atrial appendage. Thus, this proposal focuses on the obvious problems of echocardiography when assessing left atrial appendage and the role of this method in planning a potential interventional closure of left atrial appendage.

**Supplementary Information:**

The online version contains supplementary material available at 10.1007/s00392-024-02492-5.

## Introduction

The left atrial appendage (LAA) is the remnant of the embryonic left atrium (LA), while the smooth-walled LA originates from the primordial pulmonary vein and its branches. The pathogenesis of thrombogenesis in atrial fibrillation (AF) is based on the Virchow triad components leading to thrombus formation, due to endothelial irregularities, reduced blood flow velocity, and/or altered systemic hemostasis [1]. In context of thrombogenesis, endothelial irregularities have been described, particularly within the LAA and especially in AF [2].

The LAA is an actively contracting structure with muscular trabeculations forming a complex network of muscular ridges which may be impaired [3]. The shape, morphology, and fibrosis of the LAA may have an impact on reduction of blood flow velocity. The LAA is prone to stasis and is in more than 90% the most common site of thrombus formation in AF [3–5]. In initial studies, LA thrombi were found in the LAA and then grew into the surrounding LA. Consequently, the number of thrombi originating in the LAA is slightly higher than 90% [5]. However, the risk of thrombus formation in the LAA relates not only to LAA-typical properties but also to general circulatory impairments such as in conditions of a relevant reduction in left ventricular (LV) pump function [6], cardiac output (CO), and/or circulation times, which can be extremely prolonged, particularly in AF [7].

Echocardiography plays a central role in assessing the risk of embolism due to thrombus formation in the LAA [8–10]. Transthoracic echocardiography (TTE) is primarily used for analyzing the size and function of the heart cavities as well as general circulatory function in AF patients or patients with suspected AF episodes [11–13], while transesophageal echocardiography (TEE) primarily has the task of direct thrombus detection in the target structure LAA [14, 15]. The following expert proposal will address the diagnostic challenges associated with the echocardiographic assessment of LAA morphology and function. These challenges include not only the detection of thrombi but also the role of this method in planning a potential interventional LAA closure.

## Analysis of left atrial appendage (LAA) for the detection of thrombi and risk classification of LAA function: LAA flow limitations–SEC–sludge–*thrombus* formation

In computed tomography (CT) studies, four distinct main phenotypes of LAA morphology were differentiated: chicken wing, windsock, cactus, and cauliflower [16, 17]. Depending on LAA phenotype, there is a varying risk of ischemic stroke [17]. In terms of LAA morphology, LAA size, LAA orifice size, and the number of LAA lobes have an impact on blood flow alterations. Lastly, hemodynamics of LAA blood flow may be described by flow velocity and pattern, LAA emptying function, and tissue Doppler velocity with impairment promoting thrombogenesis [18]. There is growing evidence for the presence of a prothrombotic state and systemic inflammation which may affect thrombogenesis in AF, independent of LA and LAA morphology [1]. A new risk score consisting of CHA_2_DS_2_Vasc score and anatomical and geometrical complexity of LAA was associated with higher precision in predicting LAA thrombi [19]. In several analyses of LAA morphology, LAA thrombi were predominantly found in the cactus and cauliflower phenotype, whereas the chicken wing and windsock phenotypes were less strongly associated with the formation of LAA thrombi [14, 16, 20]. While by TTE LAA thrombi are generally not detected with sufficient diagnostic certainty, there are also significant challenges in TEE analysis of the LAA, e.g., distinguishing thrombi from the pectinate muscles and detecting thrombi in the shadow of the region between LAA and upper left pulmonary vein as well as the differentiation between thrombi and sludge [21–23].

The prevalence of LAA thrombi in AF patients is about 5–29% [15, 24, 25], which increases to 22–43% after recent embolic events [26–31]. Thus, an exclusion of LAA thrombi in AF patients is mandatory, especially in case of rhythm control [32, 33]. TTE has a sensitivity of only 33–72% in detecting LAA thrombi [34], whereas a sensitivity of 90–95% and a specificity of 95–100% can be achieved by TEE [27, 35–37]. TEE images should be acquired preferably in zoom settings by switching into harmonic imaging to reduce artifacts for better analysis of the LAA morphology. Biplane and 3D imaging improves the detection of LAA thrombi and supports distinguishing thrombi from LAA trabeculations and pectinate muscles [38–41]. Furthermore, size, mobility, and echogenicity of thrombi should be described [38, 40, 41].

The evaluation of the existence and the risk of developing LAA thrombi is even more challenging if spontaneous echo-contrast (SEC) and sludge are present. SEC or/and sludge is characterized by a slow turbulence reminiscent of smoke with increased echogenicity within the left atrium (LA) or LAA, which needs to be distinguished from white background noise due to excessive gain [42]. Thus, gain adjustment is necessary to reduce background noise and to facilitate a better visualization of SEC, which is more common encountered in low flow states [40, 41]. SEC is caused by ultrasonic backscatter from red blood cell aggregates seen in blood stasis or low-velocity blood flow [43]. SEC is visualized more intensively with harmonic imaging and with higher transmit frequencies compared to fundamental imaging and lower transmit frequencies. There is no universal agreement on grading LA SEC. Two different grading systems are commonly in use.

Firstly, SEC that could be just barely discerned at high gain was designated “mild,” whereas SEC that was visible at normal to low gain was designated “severe” [42].

In contrast to this simple classification, a more sophisticated system according to the following criteria was introduced:

0 = none (absence of echogenicity).

1 +  = mild (minimal echogenicity located in the LAA or sparsely distributed in the main cavity of the LA, may be detectable only transiently during the cardiac cycle, imperceptible at operating gain settings for two-dimensional (2D) echocardiography).

2 +  = mild to moderate (more dense swirling pattern than 1 + but with similar distribution, detectable without increased gain settings).

3 +  = moderate (dense swirling pattern in the LAA, generally associated with somewhat lesser intensity in the main cavity, may fluctuate in intensity but detectable constantly throughout the cardiac cycle).

4 +  = severe (intense echodensity and very slow swirling patterns in the LAA, usually with similar density in the main cavity) [44–46]

LAA sludge is an intracavitary echo density with viscid gelatinous qualities giving the impression of impending precipitation, but without a discrete organized mass, continuously seen throughout the cardiac cycle. It appears denser and more layered than severe SEC and often shows a meniscus at its upper edge. Sludge is independently predicted by enlarged LA area, reduced LAA emptying velocity, and reduced left ventricular (LV) ejection fraction (LVEF) and is independently associated with thromboembolic events and all-cause mortality [47–49]. Sludge can be differentiated from thrombus by changing the patient’s position from the left side to the right side [21], by boluses of isoproterenol [50] or by application of contrast agents [22]. However, both approaches are rarely applied in clinical scenarios. It should be recognized that differentiating sludge from severe SEC is to some extent arbitrary and subjective. SEC in the LAA is seen in 50% of AF patients and is associated with fourfold increased risk of embolic events and death [47, 48]. These results have been shown in a series of 272 patients who were followed for 17.5 months [47]. The 2016 EACVI/EHRA Expert Consensus Document on the role of multimodality imaging for the echocardiographic evaluation of AF patients [51] ranks imaging with TEE as the most reliable method to exclude LAA thrombi (negative predictive value: 100%, positive predictive value: 86%—and might be further increased by using contrast agents).

A thrombus is characterized by its visualization at least in two different planes and has an echogenicity and/or mobility that differs from the adjacent wall of the LAA. Difficulties in LAA thrombus detection include difficult-to-see side lobes and the demarcation of smaller thrombi in the LAA apex or between the pectinate muscles. In these cases, three-dimensional (3D) TEE imaging of the LAA can be advantageous, as the entire lumen of the LAA is visible in all sectional planes in postprocessing can be analyzed [14, 45]. Depending on the TEE image quality of the LAA, image acquisition should be performed both, with harmonic imaging and high penetration, but limited spatial resolution as well as with fundamental imaging and lower penetration, but high spatial resolution. Another problem in the visualization of LAA thrombi is the differentiation of larger—rather older, thrombotic structures—from the atrial wall, since almost the same echogenicity is sometimes present. In addition, a pericardial rim in the apex sometimes mimics a thrombus, but this can be clarified using contrast echocardiography [39, 52–54]. Older thrombi exhibit a higher echogenicity whereas thrombi that have not yet been fully organized often show an iso- to hypoechogenic structure. Sometimes they can only be recognized as a filling defect within the surrounding SEC. According to the recommendations of the British Society of Echocardiography [52], contrast agents should be considered in AF patients prior to rhythm control, whenever the LAA has significant SEC or cannot be adequately visualized during unenhanced TEE (opposed to recommendations of the American Society of Echocardiography where it is stated as class IIa, level of evidence B-NR) [55]. However, the highest imaging quality can only be achieved by using power modulation mode with low mechanical index (< 0.2) that is not provided by all vendors. Compared with contrast-enhanced TTE, slightly larger boluses and earlier re-injections of contrast agents might be helpful. The focus is set at the level of the farthest point of the LAA. In case of AF and highly reduced flow velocities, the time necessary to complete filling of the LAA after the application of contrast agents may be extended from a few up to 45 s. After complete LAA filling, a thrombus is characterized as a filling defect within the LAA. AF is often accompanied by a small pericardial effusion that appears as a filling defect surrounding the LAA, while both filling defects can be easily differentiated by careful scanning of the LAA using different planes.

The assessment of the LAA flow velocity by pulsed wave (pw) Doppler is an established approach to estimate the risk of thrombus formation [56, 57]. Recently, speckle tracking of LAA in a small cohort of 35 patients appeared to be promising in detecting the mobility of thrombi and as a result the associated embolic risk [58]. In a further study, LA-strain parameter showed a significant correlation with the blood flow between the LA and LAA. Further larger studies are still needed to validate the importance and the role of LAA strain in detecting thrombi and developing scores to estimate the associated embolic risk [58, 59].

TTE and TEE are of fundamental importance in the indication, planning, and implementation of electrical or pharmacological rhythm control. The detection of pathological findings—and especially the detection of LAA thrombi—can be easily and quickly diagnosed to introduce further treatment options. Tissue velocity imaging can be used for functional quantitative LAA analysis and thrombus identification [60, 61]. In addition, the color-tissue Doppler can be used for LAA thrombus detection if the Nyquist limit is chosen appropriately, since the inertia of the thrombus compared to the LAA wall movement means that different color staining of these structures can be observed during the flow of blood into and out of the LAA. This method is similar to contrast-enhanced tissue Doppler imaging [53]. However, a suspected thrombus by color-tissue Doppler should be verified by contrast imaging. For safety reasons, a TEE should be performed before rhythm control, especially in cases of inadequate anticoagulation or unclear duration of ongoing AF [62]. Visualizing LA thrombi represents a contraindication to perform cardioversion due to the direct procedural triggering of arterial embolism, notably stroke. In this context, it must be mentioned that the absence of a thrombus before rhythm control is not associated with the risk of thrombus formation due to LA stunning after successful cardioversion into sinus rhythm or after successful AF ablation. Previous studies have supported the usefulness and safety of the TEE-guided approach to cardioversion in AF patients of prolonged AF duration (> 48 h), whereas no data exist on the usefulness of TEE in short-term AF (< 48 h) [63–65]. In AF patients undergoing cardioversion, contrast-enhanced TEE images are more interpretable, if there is no SEC. In this situation, the reliability of detecting thrombi with contrast echocardiography decreases again, as contrast artifacts may appear that can be easily confused with thrombi. In the presence of SEC, contrast imaging helps to exclude thrombi and in this scenario may reduce the rate of embolic adverse events [66].

## Risk classification of LAA function for the occurrence of thromboembolic events

In principle, LAA function can be analyzed using pw Doppler echocardiography to assess LAA blood flow velocities [19, 56, 57] and tissue velocity to analyze LAA wall motion [20, 67]. The protruding shape of the LAA bulge forms a dead-end for blood flow, which is associated with low blood flow velocities, especially in LA and LAA dysfunction [21, 49, 67]. As blood flow velocities in the LAA correlate with its morphology, it is understandable that LAA bulges with a pronounced tendency to stagnate blood have a preferential propensity for thrombus formation [22, 26, 50]. Impaired LV filling in diastolic dysfunction (DD) as well as loading conditions of the LAA itself also promotes thrombus formation in the LAA [52, 55].

In addition, the degree of LAA dysfunction can be predicted by characterization of LA function [58, 59]. Despite numerous studies concerning the prognostic value of LAA flow velocities [19, 49, 56, 57], there are only a few recommendations regarding methodological aspects for determination of LAA flow velocities [62]. In principle, regional flow disparities within the LAA have been reported in the literature [63, 64]. Most important, the assessment of maximum flow velocities is used to avoid wall motion artifacts. As artifacts are more likely to be observed in the distal parts of the LAA, the maximum LAA flow velocities can preferably be derived in the proximal third of the LAA [62]. Due to improvements in ultrasound technology and the different LAA blood flow velocities due to different LAA morphology, it seems advisable to measure LAA blood flow velocities in several LAA location, e.g., in the proximal as well as in the distal part of the LAA—especially in LAA morphologies with eccentric apex formations and side lobes [64]. However, it must be ensured that the sample volume of the pw Doppler is within the LAA lumen during the entire cardiac cycle. The cut-off value of the maximum antegrade LAA velocities to predict maintenance of sinus rhythm is reported to be 0.4 m/s measured in the proximal LAA [19, 44, 57]. Lower LAA flow velocities are associated with an increased risk of LAA thrombus formation [22, 49, 52].

Similar to the analysis of the LAA flow profiles, the pulmonary venous inflow provides information about the impairment of LA filling—predominantly in sinus rhythm—as well as the potential risk of the occurrence of new or frequent AF episodes [68, 69]. During episodes of sinus rhythm in paroxysmal AF or in sinus rhythm early after cardioversion in persistent AF, the pulmonary venous flow (PVF) profile shows an increase in the atrial reflux signal, which can be explained by the intermittent impairment of LA function, the so-called LA stunning [69]. Thus, the augmentation of pulmonary vein backflow velocity during LA contraction is known as a feature responsible for AF progression [70]. In AF, atrial reverse flow disappears in the pulmonary venous flow profile. In addition, a reduction of systolic PVF can be observed by reduced systolic velocity time integral (VTI) and a reduced systolic fraction of PVF, which are both associated with reduced LAA flow, LA SEC, and the frequency of AF episodes [68]. These changes of PVF patterns, which can also be detected at early stages of AF history, cause a pathological expansion of the pulmonary veins and the LA, which therefore chronically leads to an enlargement of both cardiac structures detectable by echocardiography [71, 72].

**Fig. 1:**
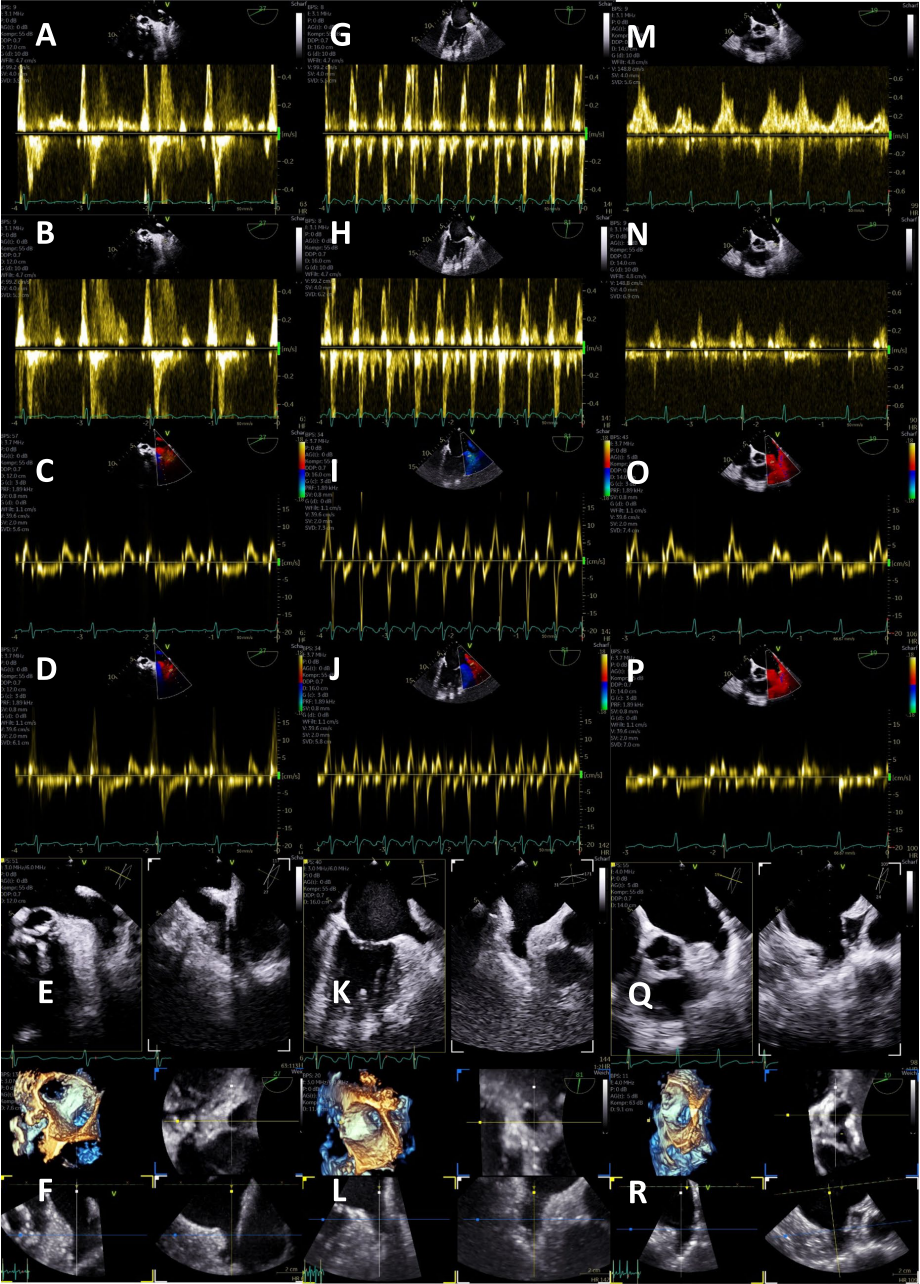
Echocardiographic analysis of the left atrial appendage (LAA) function in patients with atrial (AF) or at risk of AF. Findings of a patient with sinus rhythm (SR) and normal LAA function characterized by higher tissue Doppler velocities in the lateral than medial LAA wall—**A** LAA velocities at the proximal third of the LAA, **B** LAA velocities at the distal part of the LAA, **C** pulsed wave (pw) tissue Doppler velocities of the medial LAA wall, **D** pw tissue Doppler velocities of the lateral LAA wall, **E** biplane display of the LAA, **F** three-dimensional (3D) data set of the LAA for thrombi detection. **G**–**L** Corresponding illustrations in a patient with atrial flatter and reduced LAA function characterized by lower tissue Doppler velocities in the lateral than medial LAA wall. **M**–**R** Corresponding illustrations in a patient with AF and reduced LAA function

**Fig. 2:**
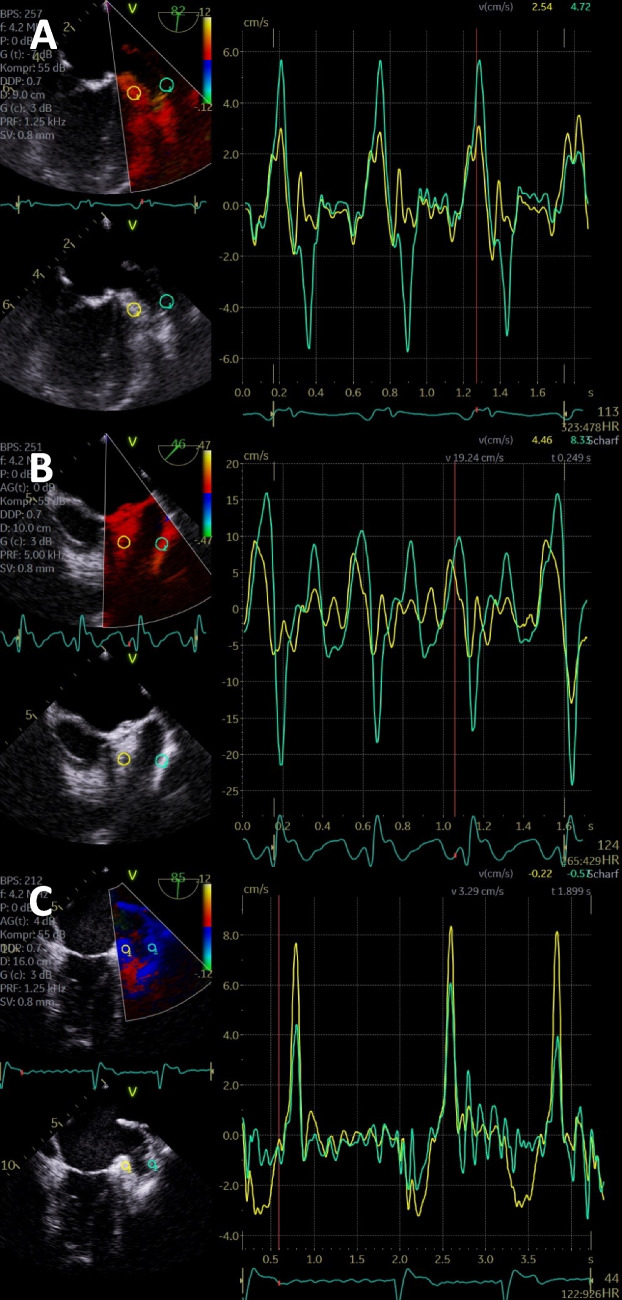
Echocardiographic analysis of the left atrial appendage (LAA) function in patients with atrial fibrillation (AF) or at risk of AF—comparison of simultaneous medial and lateral LAA pulsed wave (pw) tissue Doppler velocities by postprocessing. **A** Patient with sinus tachycardia. Medial LAA wall velocity is displayed by the yellow curve (region of interest). Lateral LAA wall velocity is displayed by the green curve (region of interest); lateral amplitudes are higher than medial reflecting normal LAA function. **B** Patient with atrial flutter (124 bpm). Medial LAA wall velocity is displayed by the yellow curve (region of interest). Lateral LAA wall velocity is displayed by the green curve (region of interest). Lateral amplitudes are higher than medial reflecting normal LAA function. **C** Patient with AF (44 bpm). Medial LAA wall velocity is displayed by the yellow curve (region of interest). Lateral LAA wall velocity is displayed by the green curve (region of interest). Lateral amplitudes are lower than medial reflecting pathological LAA function

**Fig. 3:**
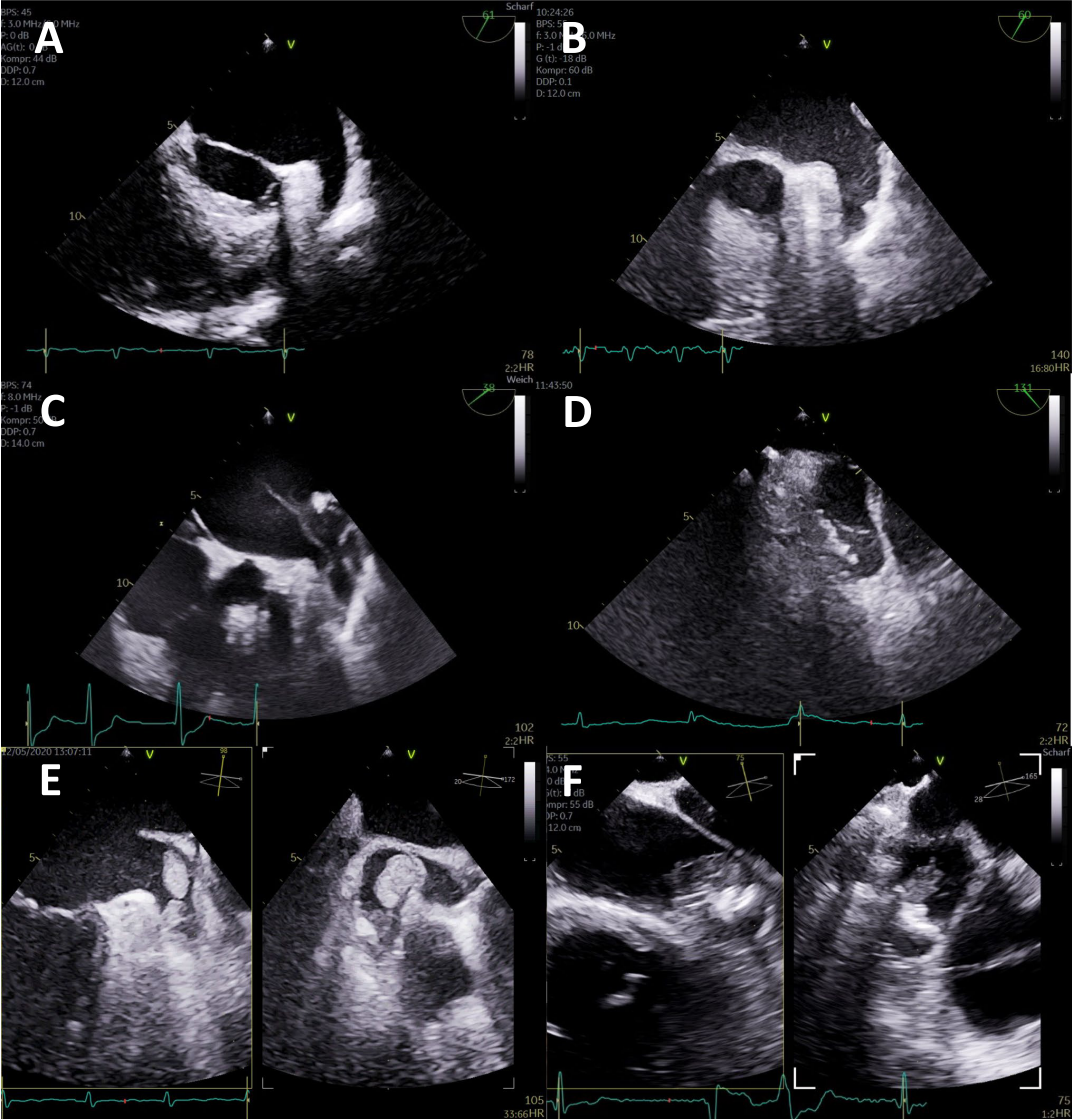
Echocardiographic analysis of left atrial appendage (LAA) for thrombus detection. **A** Oblique—chamber view of LAA with normal LAA; **B** corresponding view with low degree of spontaneous echo contrast (SEC); **C** corresponding view with progressive degree of SEC shown by the formation of streaks; **D** corresponding view with severe degree of SEC shown by the formation of powder folding in the LAA apex; **E** biplane views of large thrombus formation in the LAA; **F** biplane views of large thrombus formation on a LAA closure device

A relatively robust method to analyze LA and LAA function is the pw tissue Doppler and appears to have prognostic implications [73–76]. LAA function can be estimated by assessing regional disparities of LAA wall motion using TEE [76]. The comparison of lateral and medial LAA wall velocities seems to be an interesting parameter to characterize LAA function. In sinus rhythm as well as in AF, the lateral LAA wall velocity is higher compared to the medial LAA wall velocity, if LAA function is normal or preserved. Since the orientation of the central LAA axis can significantly influence the Doppler angle, the assessment of qualitative changes of the ratio between the regional LAA wall velocities might be better than defining absolute alphanumeric normal ranges and cut-off values for the maximum regional LAA velocities [76]. The more frequent AF episodes in paroxysmal AF, the lower the lateral LAA wall velocity. Therefore, the ratio of lateral to medial LAA wall velocity, with lower lateral than medial LAA tissue velocity, appears to be a marker of LAA dysfunction in both, patients with SR and during AF. A medial > lateral tissue velocity ratio may therefore help to identify patients with paroxysmal and still undiagnosed AF [76]. Representative illustrations of echocardiographic LAA analyses are shown in Figs. 1, 2, and 3.

## Impact of echocardiography on interventional closure of the left atrial appendage (LAA)

Percutaneous LAA occlusion (LAAO) may be considered according to current guidelines in patients who have AF and a contraindication to oral anticoagulation (IIb recommendation, level of evidence B) [32, 77]. Two strategies are currently available to exclude the LAA from the systemic circulation. First is LAAO by implanting an intracardiac device percutaneously through a venous access including transseptal puncture (TSP) to gain access to the left atrium and the LAA. Second is LAAO by using an extracardiac pericardial approach to apply an external ligature. Based on the conceptual occluder design, intracardiac devices can be further differentiated into “plug devices” and “disc-lobe devices.” In “plug devices,” the anchoring mechanism is located within the LAA and the sealing of the LAA is provided by a single component. In “disc-lobe devices,” a distal device component (a lobe or umbrella with hooks/anchors) acts as the anchoring mechanism and a separate proximal component (a conformable disc or a cover) ensures that the full cross-sectional ostial area of the LAA is sealed. A central waist connects both components. Knowledge of the different devices and their characteristics is crucial for adequate patient selection and successful guidance of LAAO procedures (Supplementary Table 1 and Fig. S1).

**Fig. 4:**
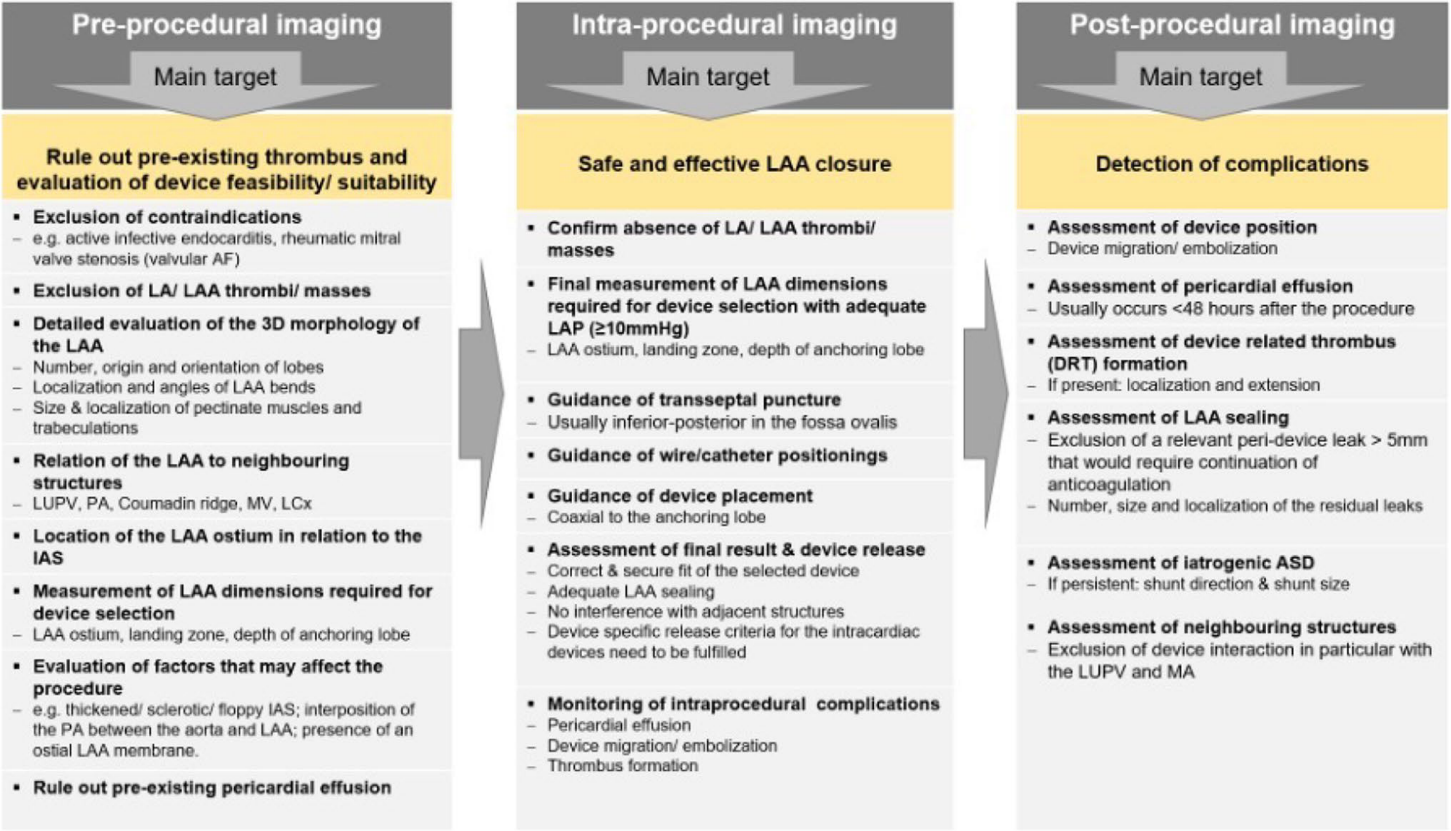
Main echocardiographic imaging tasks during left atrial appendage occlusion. AF = atrial fibrillation; LA = left atrium; LAA = left atrial appendage; LUPV = left upper pulmonary vein; PA = pulmonary artery; MV = mitral valve; LCx = left circumflex coronary artery; IAS = interatrial septum; LAP = left atrial pressure; ASD = atrial septal defect

**Fig. 5:**
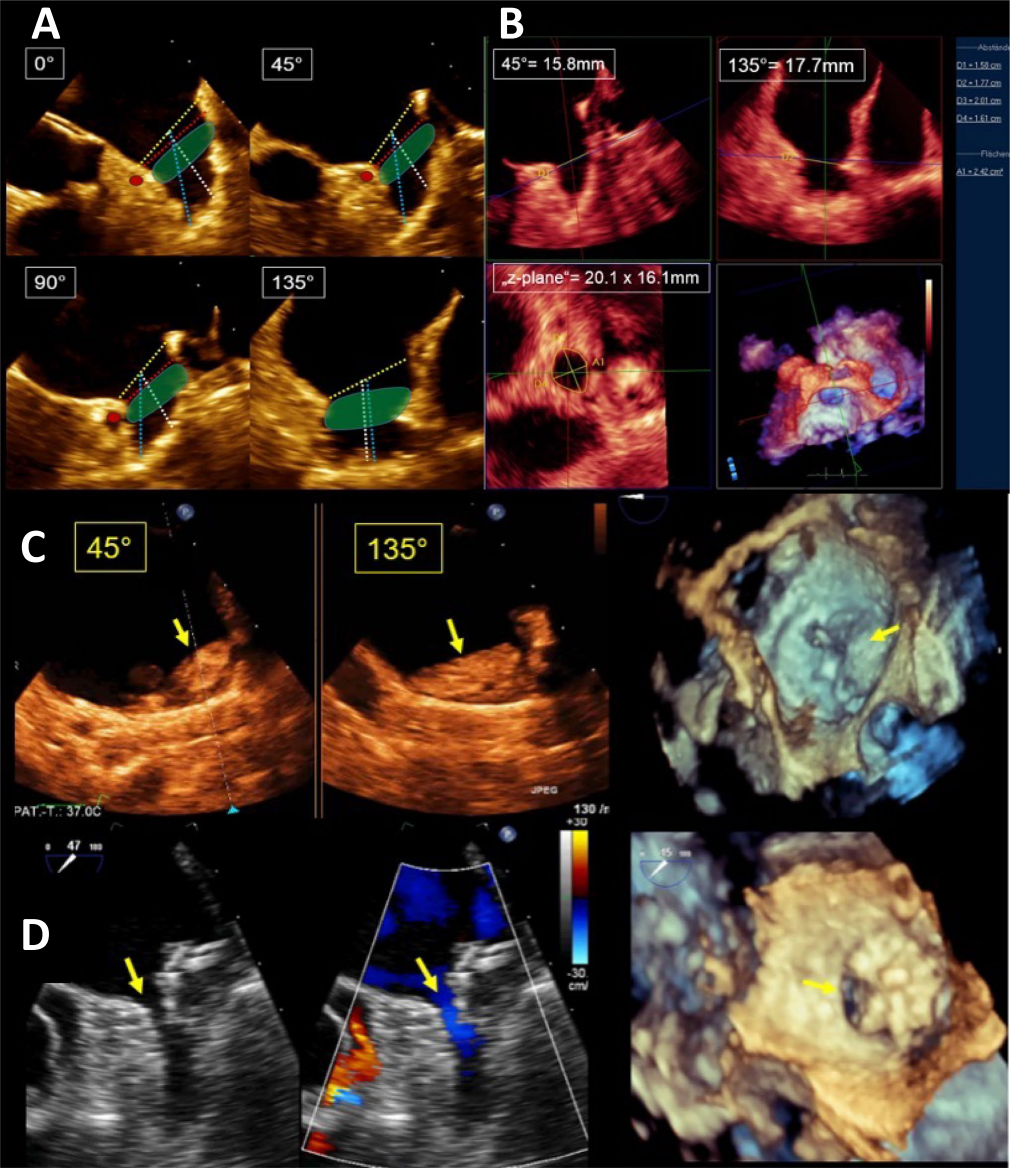
Device sizing and complications. **A** *Sizing in different 2D planes*. Using 2D transesophageal echocardiography (TEE) measurements are usually performed in at least 4 different planes as demonstrated in this example and differ slightly for the different devices. When a lobe-disc device is used, the ostial plane is usually measured in TEE from the level of the left circumflex coronary artery (LCx) (red dot) to the tip of the Coumadin ridge (yellow asterisk) (yellow dotted line) unless the Coumadin ridge is very long and prominent as it is intended to cover the entire ostial area with the disc/cover. The true anatomical ostial plane is usually located more distally and has therefore smaller dimensions (red dotted line). Landing zone dimensions are measured either at the anatomical ostial plane (from the level of the LCx (red dot) to a point approximately 10–20 mm lower than the Coumadin ridge [79] (yellow asterisk); red dotted line) or up to 10 mm deeper in the LAA cavity as required for the selected device type (green area). The length is usually measured in the axis of the device perpendicular to the landing zone (white dotted line). The total length of the LAA is measured along the dotted blue line. When multiple landing zones are conceivable, measurements should be performed for each possible landing zone as device implantation strategies may change during the procedure. **B** *Example of a 3D measurement of a device landing zone*. Multiplanar reconstruction is used to optimize sizing. The cropping lines (green and red lines in the upper images) are aligned to the main axis of the LAA, and the blue line marks the selected landing zone for the device. At the level of the blue line, a “z-plane” is created (bottom left) which allows for precise enface measurements of the landing zone. 2D TEE frequently misses the largest diameter as demonstrated in this example. Thus, 3D measurements are preferred. **C** Device-related thrombus (yellow arrows) on an Amulet occluder. **D** Peridevice leak due to an uncovered lobe (yellow arrow) after implantation of a Watchman occluder

The main periprocedural imaging requirements for LAAO are summarized in Fig. 4 according to the different phases during LAAO as outlined below. Prior to an LAAO procedure, TEE is indicated to exclude contraindications—especially LAA thrombus—to decide whether LAAO is generally feasible and which is the most suitable device type and size. For this purpose, LAA morphology, LAA dimensions, and the relationship to adjacent structures must be evaluated in detail (Fig. 4, left column). 2D and 3D TEE are the most important and most commonly used imaging modalities to support fluoroscopy during each individual procedural step [78, 79] (Fig. 4, middle column). A key TOE imaging task is to adequately measure the LAA dimensions to assess the appropriate device size (Fig. 5A, B). LAA undersizing carries the risk of device migration or embolization and may favor peridevice leakages, while oversizing may probably lead to LAA perforation, pericardial effusion, and cardiac tamponade. Aspects that need to be considered for LAA measurements are summarized in Table 1. Furthermore, monitoring of potential complications is essential throughout the entire procedure. After LAAO, TTE should be performed prior to discharge to ensure stable device positioning as well as to exclude pericardial effusion (Fig. 4, right column). The incidence of pericardial effusion occurring during the hospital stay and requiring intervention was about 2% [79].

**Table 1:** Aspects that need to be considered for LAA analysis by TEE

▪ Required measurements differ for each device type (see Suppl Tab. 1) and should be performed according to the instructions for use for the selected device.	
▪ Plug devices should have measurements at the landing zone (LZ) whereas disc-lobe devices need measurements at the LZ and additional sizing for the disc/cover. It must be ensured that the disc/cover size is big enough to seal the LAA ostium/ostial area.	
▪ Measurements should be obtained at the end of LV systole (LA diastole) when the LAA dimensions are largest.	
▪ The fluid and volume status needs to be considered to avoid undersizing (LA pressure ≥ 10 mmHg).	
▪ 2D TEE measurements should be performed in at least 4 different planes: ~ 0°, 45°, 90°, 135° [5]. The LAA orifice is most commonly oval in shape. Larger orifice diameters can be found on 120–135° planes rather than at 45 or 90°.	
▪ The device is usually selected according to the largest LAA orifice measured diameter.	
▪ Consider perimeter/area derived diameters in case of very eccentric LAA’s to avoid oversizing.	
▪ When more than one LZ is conceivable measurements should be performed for all potential LZs as strategies may change during device implantation.	
▪ The depth should be measured in the main anchoring lobe in the axis of the selected device (perpendicular to the LZ).	
▪ 3D TEE measurements correlate well with computed tomography (CT) measurements and are preferable as 2D TEE measurements tend to undersize LAA dimensions.	
▪ 3D measurements should be obtained by using 3D cropping tools which allow the precise definition of the LZ plane (direct 3D planimetry is not recommendable).	
▪ Since a deeply implanted device is a predictor for the development of a device related thrombus (DRT), LAA devices should be implanted as proximally as possible and measurements should be taken accordingly.	

There is no consensus on when to perform follow-up TTE/TEE after LAAO. However, investigators perform a TEE or alternatively a computed tomography (CT) scan 6 to 24 weeks after the procedure primarily to assess for device-related thrombus (DRT) and peridevice leaks [1, 79] (Fig. 5C, D). A reasonable compromise seems to be performing a TEE 3 months after the procedure. The incidence of DRT is between 2 and 4% [1], and DRT was an independent predictor of stroke or TIA [80, 81]. Since about 18% of DRTs develop after the 6th month [81], another control after 12 months might be beneficial [79]. Significant peridevice leakages of > 5 mm which requires continuation of anticoagulation is indicated by 1% (Amulet)–3% (Watchman) [79].

If there are no complications 12 months after the device has been implanted, further monitoring by TTE is usually performed every 1–2 years. TEE should be performed in case of abnormalities detected by TTE or clinical events, e.g., a transient ischemic attack/stroke/ischemic episode. During each follow-up, the LAAO device should be re-evaluated to ensure a stable position, absence of device migration/embolization, erosion, DRT, residual leaks, and any interference with surrounding structures (namely, the MV and the left upper pulmonary vein). In addition, the iatrogenic atrial septal defects (ASD) secondary to the TSP should be reassessed. These are usually slit-like and small and do not require closure. Most of these defects close spontaneously with only 11% of patients having residual ASD after 6 months and 7% after 1 year, respectively [82] (Fig. 4, right column).

## Summary and conclusions

This expert proposal addresses the echocardiographic challenges in assessing of LAA, which plays a crucial role in patients with proven or suspected AF. In suspected AF especially, the blood flow profiles in the LAA and in the pulmonary veins as well as the local tissue Doppler LAA profiles characterize the LAA function and allow important conclusions to be drawn about the risk of potential LAA thrombus formation and the recurrence of AF. In the presence of AF—especially prior to rhythm control—the reliable detection of thrombi as part of the therapeutic measures is necessary. However, thrombus detection in AF patients remains difficult due to the low LAA flow velocities as well as frequently occurring SEC or sludge, and therefore, contrast echocardiography should be performed if there is any doubt. In addition, this expert proposal emphasizes the utilization of echocardiography to guide the decision-making process in support of interventional closure of the left atrial appendage (LAA). Thus, it describes the necessary echocardiographic measurements of the LAA anatomy before an interventional LAA closure, considering the various commercially available occluders. In addition, monitoring during LAA closure and echocardiographic detection of postinterventional complications are described.

The complexity of this echocardiographic topic underlines the need for intensive echocardiographic training regarding the specific diagnosis and treatment of AF patients.

## Supplementary Information

Below is the link to the electronic supplementary material.

Supplementary file1 (DOCX 18.5 KB)

Supplementary file2 (DOCX 14.3 KB)

Supplementary file3 (PDF 116 KB)

## Appendix: conference discussion

Dr. Stephan Stöbe (Leipzig): The echocardiographic assessment of atrial, ventricular and valvular function in AF patients was a central topic at the “Deutscher Echokardiographie Kongress 2023” in Leipzig. Therefore, this year a second expert proposal focus on echocardiographic assessment of LAA morphology and function. ***To start this part of the conference discussion – What do you think are the important requirements for these echocardiographic tasks?***

Prof. Dr. Andreas Hagendorff (Leipzig): These echocardiographic challenges cannot be overcome by a novice. Together with methodological and technical requirements for the usage of the ultrasound machine - including a comprehensive and complete TTE as well as knowledge of LAA visualization using contrast echocardiography – the understanding of the underlying pathophysiology and clinical features of the diseases in the context of AF is necessary. Therefore, these comments on echocardiography of the LAA are certainly a valuable contribution to the current literature. ***Normally, the LAA is considered as the predominant site of origin of thrombus formation in AF. Do you consider other factors, which play an important role for thrombus formation in other regions of the LA, and what are these possible predominant LA locations?***

Ertunc Altiok (Aachen): Thrombus formation in LA regions other than the LAA can also be caused, e.g. by endothelial injuries, regional inflammation, and scars after surgical or interventional procedures. Another possible location of uncommon thrombus formation is the left atrial protrusion between the interatrial septum primum and secundum in the presence of a septal pouch. ***The interaction between LAA morphology and LAA function appears to be extremely important for thrombus formation in the LAA. However, rather than the LAA types described, aren't the individual lobe locations with possible dead water zones probably much more important than the morphological classification given in the literature?***

Tarek Bekfani (Magdeburg): Bending lobes with consecutive dead water zones are certainly conceivable in all LAA morphologies. Therefore, regardless of the morphological LAA type, a conscious search must always be made for thrombi in atypical sacs using TEE. ***In this context, the analysis of LAA function plays a crucial role. However, regarding local zones of blood stasis in lateral lobes, does it not seem any better - contrary to the guideline recommendation to determine the LAA flow velocities in the proximal third of the LAA – to analyze the individual dead water zones in the distal LAA area - especially with small sample volumes using pw Doppler - and to determine the LAA flow velocities at multiple locations?***

Dariush Haghi (Ludwigshafen): The extent to which such regions of blood stasis can be analyzed using pw Doppler remains questionable, as these LAA regions can often only be measured with an unfavorable Doppler angle. However, pw Doppler measurements at different LAA regions seems to be one approach to detect lobes with low flow conditions. ***Given the difficulties of characterizing LAA function, you introduced the simple parameter of the medio-lateral tissue velocity ratio in the LAA into the literature. Do you think this parameter will also have prognostic implications?***

Stephan Stöbe (Leipzig): Retrospectively, our own study data of the medio-lateral tissue velocity ratio in the LAA correlated with the frequency of neurological events. To clarify the predictive value of this parameter, we are currently conducting a prospective study. ***The difficult task of LAA thrombus detection in TEE - especially in SEC and sludge - can be possibly solved by contrast echocardiography. Since special technical settings are necessary for good thrombus visualization in the LAA with contrast, shouldn't such settings be permanently integrated into the ultrasound machines by the vendors?***

Andreas Helfen (Lünen): The visualization of SEC and sludge depends on imaging modalities (e.g. harmonic imaging and higher transmit frequencies compared to fundamental imaging and lower transmit frequencies.) Special settings are also necessary when using contrast echocardiography for LAA thrombus detection. Since these are vendor-dependent, a pre-made pre-set for the best possible LAA thrombus visualization would certainly be desirable for each vendor. ***According to guidelines, TEE before cardioversion is indicated for stable AF patients without previous or unclear anticoagulation. Considering possible interventions, one should evaluate directly LAA morphology for LAA occlusion in each of these TEE investigations?***

Aydan Ewers (Bochum): The aim of TEE in these patient population is to assess the presence of atrial thrombi. Of course, evaluating the morphology of the LAA during TEE is important. However, LAA occlusion may be considered according to current guidelines only in patients who have AF and a contraindication to oral anticoagulation (IIb recommendation, level of evidence B) or just a high bleeding risk. For this reason, it is sufficient to analyze LAA morphology in selected patients preferably using 3D-TEE. ***What additional benefit for LAA imaging does 3D echocardiography offer in pre-interventional TEE diagnostics?***

Karsten Lenk (Leipzig): That's a simple question. Using 3D TEE, any special features of the LAA morphology can be highlighted with good images. This is particularly important for the interventionalist when lateral lobes near the LAA ostium are detected, which can often be overlooked in monoplane or biplane imaging. ***With respect to the special importance of pre-interventional LAA diagnostics and the difficulties of monitoring during LAA interventions, courses to obtain specialist knowledge in “interventional echocardiography” have recently been introduced. In your opinion, are these modalities sufficient or would more internships lasting several weeks be a better alternative?***

Nina Wunderlich (Langen): In my opinion, a combination of specialized courses in interventional echocardiography and practical internships lasting several weeks would be the most beneficial approach. While courses provide valuable theoretical knowledge and foundational understanding, practical experience gained through internships offers hands-on training and exposure to real-world scenarios. This combination allows professionals to apply theoretical concepts in a clinical setting, refine their skills, and develop confidence in performing pre-interventional LAA diagnostics and monitoring during LAA interventions effectively. Therefore, integrating both modalities can provide a comprehensive and well-rounded education for healthcare professionals seeking expertise in this specialized field. **However, to finalize our discussion what is the most important message of the present expert proposal for echocardiographic LAA analysis?**

*Andreas Hagendorff (Leipzig):* As mentioned at the beginning of the conference discussion, the person analyzing LAA morphology and function must know all the dodges and tricks of echocardiography. Otherwise, important findings may be missed, leading to serious complications, particularly strokes and other peripheral embolisms. In addition, the screening and planning of interventional LAA closure also places demands on echocardiography in theory and practice, although the latter - as already mentioned - cannot be conveyed well in lectures and courses, but only through direct one-to-one supervision during numerous interventions on the patient.
