# Supplementary material for: Echocardiographic assessment of left atrial appendage morphology and function—an expert proposal by the German Working Group of Cardiovascular Ultrasound
Source: Clin Res Cardiol. 2024 Aug 28;114(1):25–40. doi: 10.1007/s00392-024-02492-5 (PMC11772409; doi:10.1007/s00392-024-02492-5)
Supplement: Supplementary file 1 — Supplementary file1 (DOCX 18.5 KB) [file 392_2024_2492_MOESM1_ESM.docx]

Supplementary Table 1: CE (Conformité Européenne) marked percutaneous LAA closure devices and device characteristics

| Device approach | Intracardiac | | | | Extracardiac |
| --- | --- | --- | --- | --- | --- |
| Access way | Transseptal puncture | | | | Pericardial access and transseptal puncture |
| Device concept | “Plug devices” | | “Disc-lobe devices” | | Suture based |
| Device | Watchman FLX  (Fig. 1A ) | WaveCrest*  (Fig. 1B) | Amulet  (Fig. 1C) | LAmbre  (Fig. 1D) | LARIAT Suture Device  (Fig. 1E-G) |
| Device description | Self-expanding nitinol frame with a porous PET covering > 50% of the height of the device | Self-expanding nitinol frame with an occlusive, non-thrombogenic ePTFE-material facing the LA side.  A polymer foam around the device faces the LAA side. A distal contrast injection allows for a further assessment of device stability and LAA occlusion | Nitinol wire mesh self-expanding device with a distal  lobe to anchor the device and a proximal disc to cover the ostium (each with a Dacron patch inside). A flexible waist connects both components | Ntinol frame with 2 layers of PET fabric in a proximal cover and a distal umbrella to anchor the device. Both components are connected by a central waist | The LARIAT system consists of a collapsible snare with a pre-tied suture loop that is guided epicardially over the LAA body via a magnetic epicardial/endocardial wire bridge |
| Available sizes | 5 sizes:  20, 24, 27, 31, 35mm | 3 sizes:  22, 27,32mm | 8 sizes;  16,18,20,22 (disc diameter = lobe+6mm; lobe length= 7.5mm; waist length= 5.5mm) and 25,28,31,34 (disc diameter= lobe+7mm; lobe length= 10mm; waist length=8mm) | 17 sizes:  umbrella/cover (mm):  Standard:  16/22;18/24;  20/26; 22/28; 24/30; 26/32; 28/34; 30/36; 32/36; 34/38;  36/40;  Special:  16/30;18/32; 20/32;22/34;  24/36;26/38 | 1 size:  a pre-tied 40mm suture loop |
| Anchoring mechanism | Anchoring by compression and 2 rows of 10 active fixation hooks around the perimeter of the device | 20 points of anchoring:  10 bi-directional anchors and10 single anchors located at the distal part of the device (anchors are retractable) | Anchoring by compression and small hooks around the distal part of the lobe | Anchoring by 8 small distal hooks that engage to the LAA wall, 8 bigger proximal barbs that are trapped in trabeculations and an over-sized umbrella (compression). | Suture tightening and fixation with a knot |
| Required LZ diameters | 14-31.5 mm | 14-32 mm | 11-31 mm | 14-34 mm | LAA ostium size < 40 mm (measured in CT), sizes up to 70 mm in length  can be treated. (patients with superiorly orientated LAAs and with an LAA located under a pulmonary artery cannot be closed) |
| Required length of anchoring lobe in the axis of the device | approximately half of the device diameter or longer | < 10 mm | ≥ 10 mm for 16-22 mm devices    ≥12 mm for 25-34 mm devices | ≤ 10mm | - |
| Retrievable | yes | yes | yes  (It is recommended to change the delivery sheath after a full device retrieval) | yes | yes  (retrieval may be difficult in the presence of a chickenwing LAA) |
| Repositionable | yes | yes | yes | yes | yes |
| Selection of device size | 2-4 mm larger than longest measured diameter at the LZ (oversizing up to 30 % is possible) | The longest and shortest LAA diameters are plotted on a sizing chart. The longest measured diameter should not exceed the device size. The device size should be at least 3mm larger than the average of the shortest and the longest diameter  (oversizing is not recommended) | Diameter of the lobe: 1.5 -3.4 mm larger than the longest measured diameter at the LZ.  The size of the disc needs to be larger than the LAA ostium  (relevant oversizing should be avoided) | Diameter of the umbrella: 2-6 mm (or 10-25 %) larger than the longest measured ostial diameter  (oversizing up to 40 % is possible)  The size of the cover needs to be larger than the LAA ostium | - |
| Delivery sheath sizes and configurations | 1 size: 12F;  3 different curves (Single, Double (standard), Anterior) | 1 sizes: 15F;  60° curve;  75°curve (standard);  90°curve; 90° S-curve; | 2 sizes: 12 &14 F;  TorqVue^TM^ 45° x 45° (standard) | 3 sizes: 8,9 and 10F;  Single curve 45° (standard);  Double curve 45° x 30° | Pericardial access catheter: 13F; transseptal sheath: 8F |

PET= polyethylene terephthalate; ePTFE= expanded polytetrafluoroethylene; LZ= landing zone; LA= left atrium; LAA= left atrial appendage; CT= computed tomography

*Currently not commercially available
