# Supplementary material for: Echocardiographic assessment of left atrial appendage morphology and function—an expert proposal by the German Working Group of Cardiovascular Ultrasound
Source: Clin Res Cardiol. 2024 Aug 28;114(1):25–40. doi: 10.1007/s00392-024-02492-5 (PMC11772409; doi:10.1007/s00392-024-02492-5)
Supplement: Supplementary file 2 — Supplementary file2 (DOCX 14.3 KB) [file 392_2024_2492_MOESM2_ESM.docx]

Supplement – Figure Legends

Supplementary Figure S1: Left atrial appendage closure devices: A: Watchman^TM^ device (image provided courtesy of Boston Scientific Corporation or its affiliates. All rights reserved). B: WaveCrest® device (image provided courtesy of Biosense Webster. All rights reserved). C: Amulet^TM^ occluder (image provided courtesy of Abbott. All rights reserved). D: LAmbre^TM^ device (Image provided courtesy of Lifeftech. All rights reserved). E-F: Schematic of the LARIAT^®^ suture delivery system). E: Via transseptal and pericardial access, two magnetic wires (1 and 2) are introduced, and a magnetic wire bridge is created which serves as guiderail. An endocardial compliant balloon is delivered, which is inflated at the LAA ostium to mark the “landing zone” for the suture (3). The LARIAT suture-loop (black arrow) is inserted over the wire from the epicardial side, placed around the LAA body close to the LAA ostium above the inflated balloon. F: The suture (black arrow) is then tightened by using a pre-tied one-way slip knot.

PET = polyethylene terephthalate; ePTFE = expanded polytetrafluoroethylene; LAA = left atrial appendage
