# Supplementary figures and images for: Echocardiographic assessment of left atrial appendage morphology and function—an expert proposal by the German Working Group of Cardiovascular Ultrasound
Source: Clin Res Cardiol. 2024 Aug 28;114(1):25–40. doi: 10.1007/s00392-024-02492-5 (PMC11772409; doi:10.1007/s00392-024-02492-5)

## Supplementary Figure S1:

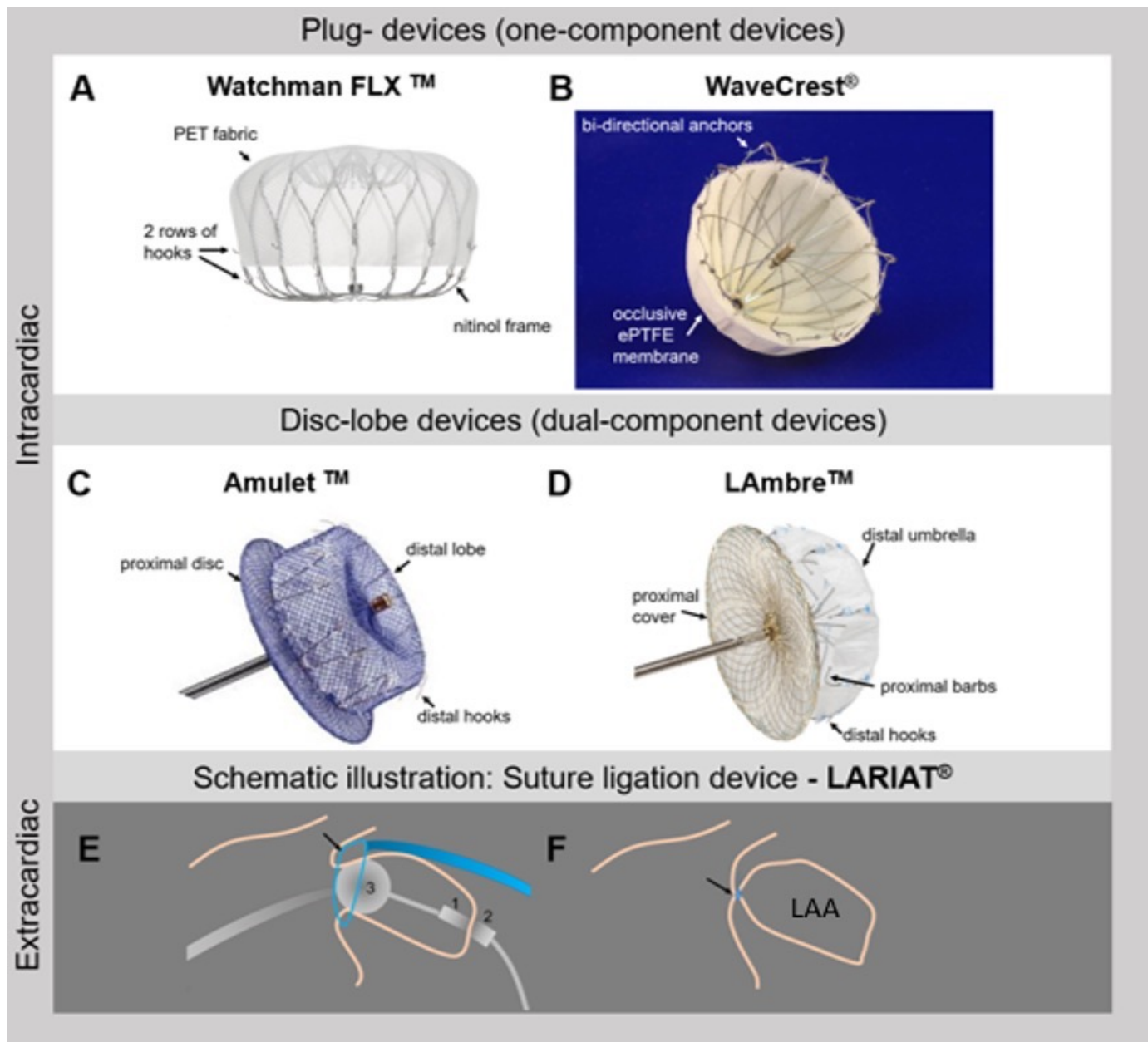

Supplement: Supplementary file 3 — Supplementary file3 (PDF 116 KB) [file 392_2024_2492_MOESM3_ESM.pdf]
